# Exploring Freeze-Drying as Strategy to Enhance Viability of *Faecalibacterium duncaniae* DSM 17677 upon Aerobic Storage and Gastrointestinal Conditions

**DOI:** 10.3390/pharmaceutics14122735

**Published:** 2022-12-07

**Authors:** Daniela Machado, Melany Domingos, Joana Cristina Barbosa, Diana Almeida, José Carlos Andrade, Ana Cristina Freitas, Ana Maria Gomes

**Affiliations:** 1 Universidade Católica Portuguesa, CBQF-Centro de Biotecnologia e Química Fina–Laboratório Associado, Escola Superior de Biotecnologia, Rua Diogo Botelho 1327, 4169-005 Porto, Portugal; 2 TOXRUN–Toxicology Research Unit, University Institute of Health Sciences, CESPU, CRL, 4585-116 Gandra, Portugal

**Keywords:** acidic pH susceptibility, aerobic storage, antioxidants, bile susceptibility, cryoprotectants, *Faecalibacterium duncaniae*, freeze-drying, gastrointestinal conditions, next-generation probiotics, viability

## Abstract

*Faecalibacterium duncaniae* is an intestinal commensal bacterium proposed as a next-generation probiotic due to its promising outcomes in the treatment and prevention of several human diseases, which demonstrate its multiple contributions to the host’s health. However, its strict anaerobic nature has created several hurdles in the development of functional foods, nutraceuticals, and biotherapeutic products. Herein, we explored freeze-dried formulations containing prebiotics, cryoprotectants, and antioxidant agents as a technological strategy to enhance the viability of *F. duncaniae* DSM 17677 upon aerobic storage and gastrointestinal tract conditions. Our results indicate that freeze-dried *F. duncaniae* in a matrix containing inulin, sucrose, cysteine, and riboflavin survived at levels higher than 10^6^ CFU/g and around 10^5^ CFU/g after 1 and 4 days of aerobic storage at room temperature, respectively. Thus, the freeze-dried formulation with inulin, sucrose, cysteine, and riboflavin presents as a protective strategy to improve *F. duncaniae* viability under aerobic environments. Nevertheless, incorporation of a suitable coating aimed at protecting *F. duncaniae* against the detrimental gastrointestinal passage effects is urgently required, given its high susceptibility to extreme acidic pH values and bile.

## 1. Introduction

In the last decades, live bacterial cells have been widely exploited, either as therapeutic agents or as carriers to deliver drugs, presenting great outcomes in the treatment of several human diseases [1,2]. Specifically, the use of live beneficial microbes, termed as probiotics, in dietary supplements, functional foods, or pharmabiotic forms, constitutes one of the most successful approaches to achieving health benefits and improving people’s welfare and quality of life [3]. Moreover, the growing knowledge regarding the key role of gut microbiota in human health has increased interest in using intestinal commensal bacteria, as well as the traditionally used lactobacilli and bifidobacteria species, as probiotics [4]. Among those, *Faecalibacterium duncaniae* (formerly designated *Faecalibacterium prausnitzii*) is one of the most promising candidates proposed as a next-generation probiotic, given its great potential to treat and prevent various inflammatory diseases [4,5,6]. More specifically, oral administration of *F. duncaniae* DSM 17677 or its supernatant displayed protective effects in the colitis mice model [7]. Furthermore, analysis of the phenotypic and genotypic antimicrobial resistance profile of the *F. duncaniae* DSM 17677 strain showed that it has a low risk of carrying acquired antimicrobial resistance. This finding is a valuable contribution for the establishment of this strain as safe for human and animal consumption, and, consequently, the finding increases the likelihood of its approval to be applied as a food or feed additive [8]. Despite the promising outcomes, the strict anaerobic nature of *F. duncaniae* has created serious technological obstacles to its cultivation and handling, consequently hampering its application in the food and pharmaceutical industries [9,10]. Envisaging its use as a probiotic, effective delivery strategies must be developed to ensure that this bacterium is maintained at high viability levels during the production process, distribution chain, and shelf-life. In addition, they must also guarantee its survival after ingestion throughout the gastrointestinal tract (GIT) passage in order to ultimately reach the colon in the appropriate amounts that are known to exert the intended positive effects on the host [9].

Encapsulation techniques are gathering the attention of industries and the scientific community as a strategy to ensure the high stability/viability of probiotic strains, which is defined as the cellular entrapment/coating within a material or mixture of materials [11,12,13]. Among the several methods that may be used to encapsulate bioactive compounds, including probiotics, drying techniques are frequently preferred because the drying process reduces the formulation’s water content, contributing to a higher stability over time [13,14,15,16]. Freeze-drying is one of the most popular drying techniques for long-term probiotic preservation [14,17].

Freeze-drying, also known as lyophilization, involves three main steps: (1) freezing of the cell culture; (2) primary drying, in which the frozen water is removed by sublimation under vacuum; and (3) secondary drying, in which the unfrozen water is removed by desorption [14]. As freeze-drying conditions are milder than those of other drying techniques, such as spray-drying, probiotic cultures dried by this technique frequently display higher survival rates [18,19]. However, freeze-drying is an expensive and time-consuming batch process, and the final product is often compact and hard, which requires an additional step to obtain individual powder particles [14,19].

During the freezing-drying process and subsequent storage, microbial cells are exposed to harsh conditions, including mechanical, osmotic, and oxidative stressors. The effect of these stressors can be minimized by the incorporation of cryoprotectants (e.g., inulin, sucrose, and trehalose) and antioxidant agents (e.g., cysteine and riboflavin) [19,20]. For instance, Khan and coworkers freeze-dried the strict anaerobic *F. duncaniae* using a formulation containing inulin, cysteine, and riboflavin and obtained around 70% survival upon 24 h of storage with ambient air [10]. Using the work of Khan et al. as a starting point, the present study aimed to explore freeze-dried formulations, combining different protective agents to improve the viability of *F. duncaniae* DSM 17677 during aerobic storage and when exposed to GIT conditions. Thus, first, we evaluated the viability of *F. duncaniae* DSM 17677 free cells when exposed to an aerobic atmosphere and to GIT conditions, specifically acidic pH values (3 and 5) and bile. Then, the impact of the freeze-drying process, using different combinations of protective agents, on *F. duncaniae* viability during aerobic storage at room temperature was evaluated. Finally, the protective effect of a selected freeze-dried formulation on *F. duncaniae* viability when exposed to acidic pH and bile was assessed.

## 2. Materials and Methods

### 2.1. Bacterial Strain and Growth Conditions

*Faecalibacterium duncaniae* DSM 17677 strain (Leibniz Institute DSMZ-German Collection of Microorganisms and Cell Cultures, Braunschweig, Germany) was used in this study. It was initially cultured according to the recommended conditions proposed by DSMZ [21]. For long term storage, this strain was kept frozen at −80 °C in sBHI broth [Brain Heart Infusion medium (37 g/L; VWR International, Leuven, Belgium) supplemented with yeast extract (5 g/L; VWR International), hemin (5 mg/L; Alfa Aesar, Kandel, Germany), vitamin K1 (5 µL/L; Sigma-Aldrich Co., St. Louis, MO, USA), and L-cysteine (2 g/L; Alfa Aesar)], as previously used by Maier et al. [22], with 20% (*v/v*) of glycerol (Fisher Scientific, Loughborough, UK). For each assay, a *F. duncaniae* glycerol stock was thawed and grown in sBHI broth for 16 h at 37 °C under anaerobic conditions (85% N_2_, 5% H_2_, and 10% CO_2_) achieved in an anaerobic incubator (Whitley A35 HEPA anaerobic workstation, Bingley, UK). Afterwards, the previously grown culture was transferred to sBHI broth (in a proportion of 1:100), and this bacterial suspension was anaerobically incubated during 10 h at 37°C for the following experiments.

### 2.2. Aerobic Environments Tolerance of Free Cells

The tolerance of *F. duncaniae* DSM 17677 free cells (non-formulated nor freeze-dried cells) to an aerobic atmosphere was measured through two approaches, namely exposing both (i) sBHI agar plates inoculated with *F. duncaniae* (cell concentration of 10^7^ CFU/mL) and (ii) 15 mL centrifuge tubes containing a bacterial suspension of *F. duncaniae* in sBHI broth (cell concentration of 10^7^ CFU/mL) for 1, 2, 3, and 5 min at ambient air (without agitation). After exposure, the plates and tubes were placed inside the anaerobic chamber. The viability of *F. duncaniae* in the plates was assessed directly by incubating the plates anaerobically at 37 °C for 48 h. For the bacterial suspensions, the viability at each sampling timepoint was assessed via colony-forming units (CFU) enumeration by plating 10 μL of decimal dilutions on sBHI agar plates, which were then anaerobically incubated at 37 °C for 48 h. The appropriate growth controls were prepared without exposure to the aerobic atmosphere and processed as mentioned for each procedure. All assays were repeated independently at least twice, and CFU plating was performed in triplicate.

### 2.3. Acid and Bile Susceptibility of Free Cells

For the acidic pH susceptibility experiments, hydrochloric acid at 6 M was added to 15 mL centrifuge tubes containing grown *F. duncaniae* cultures in sBHI broth (in a concentration of 10^7^ CFU/mL) in order to reach pH values of 3 and 5. After 1 and 2 h of exposure to acidic pH, *F. duncaniae* CFU enumeration was performed as described previously.

For the bile susceptibility assays, bile solution at 5% (*m/v*) was prepared by dissolving 0.5 g of bile extract porcine (Sigma) in 10 mL of sterile deionized water. As bile solubilization requires exhaustive mixing [23], the bile solution was subsequently placed in an orbital shaker (Bench Top Shaking Incubator, Wiggen Hauser, Berlin, Germany) at 37 °C and 200 rpm for 30 min. Afterwards, the bile solution was added to 15 mL centrifuge tubes containing a bacterial suspension of *F. duncaniae* in sBHI broth (concentration of 10^7^ CFU/mL) to reach a final bile concentration of 0.1% (*m/v*), 0.25% (*m/v*), and 0.5% (*m/v*). Cultures were incubated at 37 °C for 3 h under anaerobic conditions, with CFU enumeration being performed every hour, as described previously.

For both tests, growth controls were included without hydrochloric acid and bile solution, respectively. All assays were repeated independently at least twice and included two replicates for each pH and bile concentration tested, in which CFU plating was performed in triplicate.

### 2.4. Formulation Procedure

The formulation procedure was based on the previous work by Khan et al. [10] with some modifications. Briefly, broth cultures of *F. duncaniae* (in a concentration of 10^7^ CFU/mL) were transferred to 50 mL centrifuge tubes and centrifuged at 4470× *g* for 5 min at 4 °C. Bacterial pellets were then washed in phosphate buffer saline (PBS, VWR Chemicals, Aurora, OH, USA) and re-centrifuged in the same conditions to obtain a cell-washed pellet. Bacterial pellets from 35 mL of *F. duncaniae* broth cultures were re-suspended in one of the following, supplemented with 200 µL of 16.5 mM riboflavin (Sigma; solution prepared in PBS):

1. 400 µL of a solution containing inulin [5% (*m/v*), Orafti Beneo, Mannheim, Germany], trehalose dihydrate [5% (*m/v*), Sigma], and 0.2% (*m/v*) cysteine prepared in PBS (ITCR);
2. 400 µL of a solution containing inulin [5% (*m/v*)], sucrose [2.5% (*m/v*)], trehalose dihydrate [2.5% (*m/v*)], and 0.2% (*m/v*) cysteine prepared in PBS (ISTCR);
3. 400 µL of a solution containing inulin [5% (*m/v*)], sucrose [5% (*m/v*), Sigma], and 0.2% (*m/v*) cysteine prepared in PBS (ISCR);
4. 400 µL of a solution containing inulin [10% (*m/v*)] with 0.2% (*m/v*) cysteine prepared in PBS (ICR).

All solutions were sterilized by filtration using a cellulose acetate membrane filter (Sartorius, Goettingen, Germany) before their addition to bacterial pellets. Next, all the formulations incorporating *F. duncaniae* were homogenized and then frozen at −80 °C overnight. Frozen samples were freeze-dried for 24 h using a freeze drier (LyoQuest, Telstar, Barcelona, Spain) and stored inside a desiccator at room temperature (around 21 °C) until further analysis.

It should be noted that the bacterial cultivation, formulation procedure, and testing of tolerance to GIT conditions were conducted anaerobically, while freeze-drying and storage were performed aerobically. For each formulation and time point, two replicates were used and inoculated in triplicate in sBHI agar plates in order to characterize their impact on the viability and stability of *F. duncaniae* during aerobic storage and when exposed to acidic pH values and bile as described below.

### 2.5. Viability and Stability of Freeze-Dried Formulations during Aerobic Storage

For viability and stability assays, freeze-dried formulations incorporating *F. duncaniae* were exposed to atmospheric air at ambient temperature for 0 and 24 h. In addition, the ISCR formulation was selected to test the stability of *F. duncaniae* during a more prolonged aerobic storage period, with sampling points at 0, 24, 48, 72, and 96 h. For each sampling point, the formulations were placed back in the anaerobic chamber, rehydrated in sBHI, and then ten-fold serial dilutions were performed with PBS. Fifty µL of each dilution were plated in triplicate on sBHI agar plates that were incubated anaerobically at 37 °C for 48 h. After incubation, CFU numbers were determined, and the results were expressed as log CFU per gram (log CFU/g) for each freeze-dried formulation.

### 2.6. Viability of a Selected Freeze-Dried Formulation after Exposure to Acidic pH and Bile

The acid and bile tolerance assays were performed for the ISCR freeze-dried formulation immediately after the freeze-drying procedure (i.e., at day 0 of aerobic storage). In the acid tolerance assays, the freeze-dried formulation was initially rehydrated with 2 mL of sBHI under anaerobic conditions. Then, the pH of the rehydrated formulations was adjusted to values of 3 and 5 with 2 M HCl and incubated at 37 °C under anaerobic conditions. In addition, a growth control (without addition of HCl) was included. The number of viable cells (log CFU/g) was determined at 0 and after 2 h of exposure to the acidic pH.

The bile tolerance of the freeze-dried formulation was tested by adding bile solution to 1.8 mL of the rehydrated formulation in sBHI to reach the final bile concentrations of 0.25% (*m/v*) and 0.5% (*m/v*). Moreover, a growth control (without bile) was included. Then, aliquots were taken at 0 and after 3 h of bile exposure at 37 °C under anaerobic conditions, and cell viability (log CFU/g) was determined.

### 2.7. Statistical Analysis

Data were expressed as the mean ± standard deviation (SD) of replicates. Results from exposure to oxygen, acid pH values and bile, and aerobic storage were analyzed using the Wilcoxon signed rank test, as the data did not follow a normal distribution according to the Shapiro–Wilk test. All tests were performed with a significance level of 5% (*p* value < 0.05) using GraphPad Prism software version 5.0 (GraphPad Software, San Diego, CA, USA).

## 3. Results and Discussion

### 3.1. Oxygen Sensitivity of F. duncaniae DSM 17677 Free Cells

After the exposure of sBHI agar plates inoculated with the *F. duncaniae* DSM 17677 strain to ambient air, no viable cells could be detected after just 1 min of exposure. In contrast, *F. duncaniae* bacterial suspensions within 15 mL centrifuge tubes maintained their viability during 5 min of oxygen exposure (see Table 1). We hypothesize that the disparity of results may be explained by the difference between the surface areas exposed to the aerobic atmosphere. A thin layer of bacterial suspension spread over a wide surface area was exposed to oxygen in the inoculated plates, whereas in the approach involving the exposure of bacterial suspension within 15 mL centrifuge tubes, only the very top layer of the bacterial suspension was exposed to the aerobic atmosphere. Indeed, the diffusion coefficient for oxygen into water is very small, leading to a low permeation into the liquid culture media. In a static culture, the diffusion of oxygen into the medium only occurs at the very top surface of the liquid that is directly exposed to the atmosphere; therefore, everything below approximately 1 mm is considered to be growing under anaerobic conditions [24]. Duncan and colleagues were pioneers in demonstrating the strict anaerobic nature of *F. duncaniae* DSM 17677 (=A2-165). Their group reported that air ambient exposure of inoculated plates for more than 2 min was enough to prevent subsequent anaerobic growth [5]. The present findings are consistent with those reported by Duncan et al. and provide additional insight when evaluating *F. duncaniae* viability in broth cultures after aerobic exposure.

### 3.2. Acid and Bile Sensitivity of F. duncaniae DSM 17677 Free Cells

*Faecalibacterium duncaniae* free cells’ viability under low pH values and in the presence of bile was evaluated in order to assess the two main stressors encountered by *F. duncaniae* during gastrointestinal transit. As presented in Table 2, losses in bacterial viability higher than 4 log CFU/mL (to levels below the limit of detection) were observed just after exposure to pH 3 for 1 h. In contrast, after 1 and 2 h exposure to pH 5, *F. duncaniae* viability only underwent slight fluctuations (*p* > 0.05; see Table 2). Furthermore, *F. duncaniae* DSM 17677 free cells demonstrated a high sensitivity to bile, since cultivable cell numbers were lower than the limit of detection of the CFU enumeration technique for all times of exposure, independently of the bile concentrations (0.1%, 0.25%, and 0.5%) tested. In addition, our results are in accordance with previous reports in terms of pH values tolerated by the *Faecalibacterium* species and its sensitivity to bile. Indeed, several *Faecalibacterium* strains have been described as being able to grow at pH values ranging between 5.0 and 6.7, while the absence of bacterial growth was found at pH values lower than 4.5 and in the presence of bile at 0.1%, 0.25%, and 0.5% [25,26,27]. However, it should be noted that these previous studies evaluated bacterial growth through measurements of optical density, which is a simple, inexpensive, and quick technique, albeit less accurate because it just estimates viability. The absence of a direct correlation between CFU counts and optical density measurements in *Faecalibacterium* cultures has been previously reported [28]. Taking this into account, our results further substantiate this knowledge, as the evaluation of the extent of the effects of exposure to acid pH (pH 3 and pH 5) and bile on the *F. duncaniae* free cells viability uses the CFU enumeration technique, which provides the determination of viable and cultivable cells. Additionally, our findings highlight the need to develop a suitable delivery system for *F. duncaniae*, given its high susceptibility to oxygen and gastrointestinal conditions.

### 3.3. Aerobic Exposure of F. duncaniae Freeze-Dried Formulations at Room Temperature

**Figure 1:**
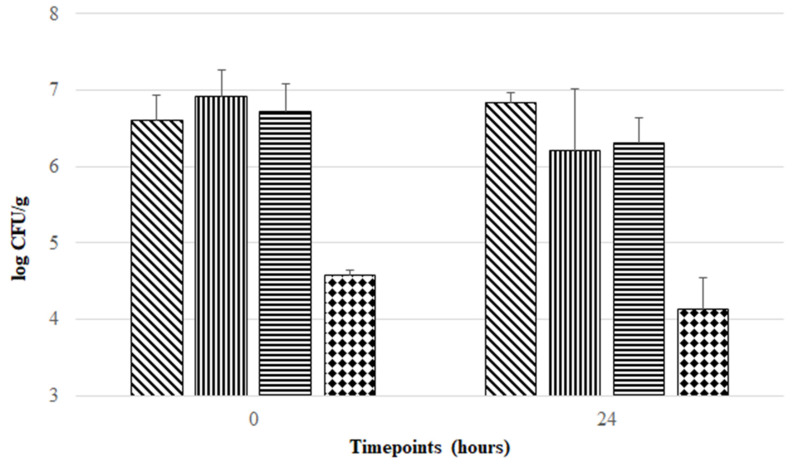
Evolution of viability of *F. duncaniae* DSM 17677 (mean values in log CFU/g) in different freeze-dried formulations (diagonal lines: ITCR; vertical lines: ISTCR; horizontal lines: ISCR; and diamond pattern: ICR) during aerobic storage at room temperature for 24 h. Error bars represent standard deviation of the mean.

Freeze-drying is a technique often used for probiotic preservation, allowing cost-effective delivery and management. However, during the freeze-drying procedure and subsequent storage, probiotic strains are subjected to stress conditions that may impair their viability and functionality [19,20]. Therefore, in order to maintain the viability of probiotic strains, exploitation of stabilizing strategies provided by cryoprotectant, prebiotic, and antioxidant compounds during freeze-drying and storage is a crucial and challenging task in the development of probiotic formulations. This rationale was taken as a starting point for the development of four preservation matrices in this study. Several studies support the use of inulin, trehalose, and sucrose as prebiotic/cryopreserving agents able to act as nutritional substrates and protecting agents during the freeze-drying procedure [20,29,30], and the use of cysteine and riboflavin as antioxidant and redox mediators [10,31]. Thus, four combinations of these agents were tested. As can be seen in Figure 1, all formulations provided protection during freeze-drying and subsequent aerobic storage at room temperature, but at different magnitudes. ITCR, ISTCR, and ISCR formulations offered higher protection during freeze-drying, maintaining *F. duncaniae* viability around 10^7^ CFU/g. In contrast, the ICR formulation presented the lowest viability for *F. duncaniae* (below 10^5^ CFU/g) in the post freeze-drying period. Although there was no statistically significant difference (*p* > 0.05) in cell viability found in each freeze-dried formulation when comparing timepoint 0 h with 24 h, Figure 1 shows that ITCR offered the highest stabilization effect (without loss in *F. duncaniae* viability, comparing the mean log CFU/g values between 0 and 24 h), followed by ISCR and ICR with viability reductions of 0.41 and 0.44 log CFU/g, respectively, when comparing mean log CFU/g values of timepoint 0 h with 24 h. In contrast, the ISTCR formulation exerted the lowest stabilization effect during 1 day of aerobic storage, with a viability reduction of 0.73 log CFU/g when comparing mean values of log CFU/g at 0 h versus 24 h.

To the best of our knowledge, only one previous study assessed the stabilizing effect of prebiotic, cryoprotectant, and antioxidant agents in enhancing *F. duncaniae* viability during freeze-drying and subsequent aerobic storage. Khan and colleagues were pioneers in this matter when they demonstrated that *F. duncaniae* DSM 17677 freeze-dried in a matrix composed of 10% (*m/v*) inulin, 0.2% (*m/v*) cysteine, and 16.5 mM riboflavin (corresponding to the ICR formulation tested in the present work) was able to survive ambient air exposure for 24 h with a percentage of survival around 70% [10]. However, these researchers did not refer to *F. duncaniae* viability in terms of CFU/g (nor did they assess viability beyond 24 h). Nevertheless, it has been reported that probiotic bacteria must be present at minimum concentrations of 10^6^–10^7^ CFU/g or CFU/mL in order to exert their positive effects [32,33]. Considering this requirement, our results suggest that the formulation proposed by Khan and colleagues (ICR formulation) does not ensure this minimum level of probiotic bacteria that should be present in probiotic products.

**Figure 2:**
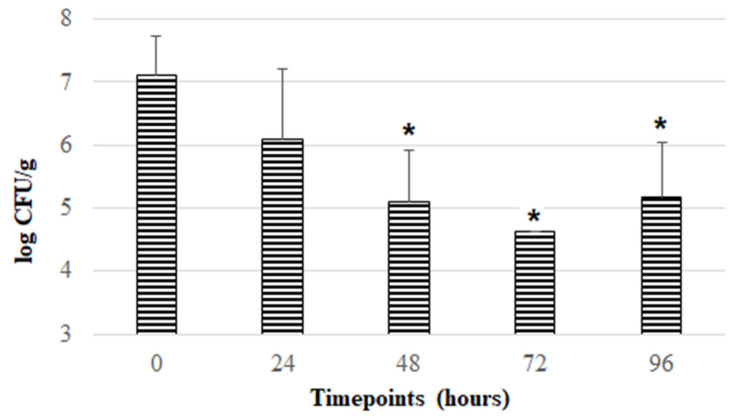
Evolution of viability of *F. duncaniae* DSM 17677 (mean values in log CFU/g) incorporated in freeze-dried formulation containing inulin, sucrose, cysteine, and riboflavin during aerobic storage at room temperature throughout 96 h. Error bars represent standard deviation of the mean. Bars marked with * represent statistically significant differences (*p* < 0.05) in comparison to data reported at 0 h.

After the initial screening, the ISCR formulation was selected to test the stability of *F. duncaniae* DSM 17677 during a more prolonged aerobic storage period, i.e., 96 h (4 days) at room temperature. This formulation was chosen because enabling high *F. duncaniae* viability and stability during 24 h of aerobic storage (with levels between 10^6^–10^7^ CFU/g) is economically more viable in comparison with the other formulations tested. As can be observed in Figure 2, a downward trend in the viability of *F. duncaniae* DSM 17677 was found throughout aerobic storage from 0 to 72 h, with a reduction of around 2.5 log cycles, reaching a mean log CFU/g of 4.62 at the timepoint of 72 h. After 96 h of aerobic exposure, viability appeared to reach a stabilizing effect, as it was maintained at levels of around 10^5^ CFU/g. Thus, the ISCR formulation containing inulin [5% (*m/v*)], sucrose [5% (*m/v*)], cysteine [0.2% (*m/v*)], and riboflavin (16.5 mM) appears to be a promising solution to enhance the survival of this strict anaerobic bacterium in aerobic environments and simultaneously protect against detrimental conditions underlying the freeze-drying procedure.

### 3.4. Exposure of F. duncaniae Freeze-Dried in Inulin, Sucrose, Cysteine and Riboflavin Matrix to Acidic pH Values and Bile

It has been indicated that probiotic strains must survive manufacture, storage, and, after consumption, the passage through the harsh GIT conditions in order to reach the colon in adequate viable cell numbers [9]. In the present work, the ISCR formulation was exposed to two of the main stressors of the digestive tract: acidic pH (pH 3 and pH 5) and bile (at 0.25% and 0.5%). As presented in Table 3, *F. duncaniae* viability decreased to undetectable levels in the freeze-dried formulation after exposure to pH 3 for 2 h or to bile concentrations of 0.25% (*m/v*) and 0.5% (*m/v*) for 3 h. However, freeze-dried bacteria when subjected to pH 5 for 2 h showed a lower reduction of viability. These results demonstrate that the ISCR formulation does not offer protection to *F. duncaniae* when exposed to pH 3 and bile concentrations of 0.25 and 0.5% (*m/v*), similar to what was verified for the free cells. In alignment with our findings, recently Raise and colleagues used one *Faecalibacterium* isolate (named *F. prausnitzii* CNCM I-4573) and demonstrated that both free and freeze-dried cells—the latter in a matrix containing sucrose (0.49 M) and cysteine (5 mM)—had a full viability loss after contact with simulated gastric fluid, containing pepsin (3 g/L) at pH 1.8, and the simulated distal jejunum buffer, containing pancreatin (5 g/L) and bile salts (3 g/L) at pH 6.8 [34]. Together, these findings corroborate that *F. duncaniae* is highly sensitive to acidic pH (equal to or lower than 3) and bile, suggesting the urgent need to develop suitable coatings to protect this bacterium from harsh GIT conditions, mainly extreme acidic pH and bile, in a similar way to previous studies involving other anaerobic beneficial bacteria [11,30,35].

## 4. Conclusions

The present study provides further robustness and brings new insights regarding knowledge of *F. duncaniae* DSM 17677 susceptibility towards environmental stresses, including aerobic atmosphere, acidic pH values, and bile. Our data indicated that the tolerance of *F. duncaniae* DSM 17677 free cells to an aerobic atmosphere was higher when the bacterium was suspended in a liquid medium rather than in inoculated sBHI agar plates, which could be explained by the low oxygen diffusion in broth cultures. Furthermore, we demonstrated that the viability of this bacterial strain was strongly impaired at pH 3 and in the presence of bile. To enhance *F. duncaniae* viability under an aerobic environment, namely acid pH and bile, a freeze-drying strategy with a combination of different protective agents was explored. Interestingly, our results showed that *F. duncaniae* freeze-dried in an inulin, sucrose, cysteine, and riboflavin matrix was able to survive at levels around 10^5^ CFU/g after 96 h of aerobic storage at room temperature. However, when this formulation was exposed to bile and acidic pH values, no further protection was granted in comparison to free cells. Therefore, future studies performing some adaptations of the freeze-dried formulation are needed in order to reach *F. duncaniae* levels higher than 10^6^ CFU/g (corresponding to the minimum level required in probiotic products) during prolonged aerobic storage. Furthermore, additional works aiming to develop an optimal coating to protect the *F. duncaniae* freeze-dried formulation from harsh GIT conditions are urgently required.

## Figures and Tables

**Table 1:** Viability of *F. duncaniae* DSM 17677 free cells when exposed to ambient air. Data represent the mean values of log CFU/mL ± standard deviation (SD) obtained in the exposure of inoculated plates and after plating of bacterial suspension within 15 mL centrifuge tubes exposed for different time periods.

Oxygen Exposure Time (min)	log CFU/mL ± SD	
	Exposure of Inoculated Plates	Exposure of Bacterial Suspensions
0	7.29 ± 0.25	7.29 ± 0.25
1	<LOD ^1,^*	7.52 ± 0.03
2	<LOD ^1,^*	7.45 ± 0.20
3	<LOD ^1,^*	7.23 ± 0.15
5	<LOD ^1,^*	7.40 ± 0.13

^1^ Limit of detection (LOD) is equal to 3 log CFU/mL according to CFU enumeration technique. * represent statistically significant differences (*p* < 0.05) between data obtained in each timepoint (1, 2, 3 and 5 min) compared to those obtained at 0 min.

**Table 2:** Viability of *F. duncaniae* DSM 17677 free cells when exposed to acidic pH. Data represent the mean values of log CFU/mL ± standard deviation (SD) obtained in growth control and after exposure to pH 3 and pH 5 for 1 and 2 h.

Exposure Time (h)	log CFU/mL ± SD		
	Growth Control	pH Values	
		3	5
0	7.35 ± 0.21 ^1^	7.35 ± 0.21	7.35 ± 0.21
1	7.43 ± 0.13	<LOD ^2,^*	7.36 ± 0.15
2	7.40 ± 0.24	<LOD ^2,^*	7.17 ± 0.19

^1^ Growth control displayed a pH value of 6.56 ± 0.03. ^2^ Limit of detection (LOD) is equal to 3 log CFU/mL according to CFU enumeration technique. * represent statistically significant differences (*p* < 0.05) in viability of *F. duncaniae* free cells exposed to pH 3 or 5 in comparison to growth control at each timepoint 0, 1, and 2 h.

**Table 3:** Acid and bile tolerance of *F. duncaniae* DSM 17677 incorporated in the ISCR freeze-dried formulation containing inulin [5% (*m/v*)]), sucrose [5% (*m/v*)]), cysteine [0.2% (*m/v*)]), and riboflavin (16.5 mM). Results were expressed as mean values in log CFU/g ± standard deviation (SD).

Exposure		log CFU/g ± SD
pH	Control before exposure (T = 0 h)	6.96 ± 1.02
	Control after 2 h	7.85 ± 0.47
	pH 3 after 2 h	<LOD ^1,^*
	pH 5 after 2 h	6.24 ± 0.97
Bile	Control before exposure (T = 0 h)	7.23 ± 0.26
	Control after 3 h	7.18 ± 0.71
	Bile 0.25% (*m/v*) after 3 h	<LOD ^1,^*
	Bile 0.5% (*m/v*) after 3 h	<LOD ^1,^*

^1^ Limit of detection (LOD) is equal to 4 log CFU/g according to the enumeration technique. * represent statistically significant differences (*p* < 0.05) between data involving exposure to each acid pH values (3 and 5) for 2 h or bile exposure during 3 h versus respective control.

## Data Availability

Not applicable.
